# Seafood Consumption and Components for Health

**DOI:** 10.5539/gjhs.v4n3p72

**Published:** 2012-05-01

**Authors:** Ryota Hosomi, Munehiro Yoshida, Kenji Fukunaga

**Affiliations:** 1 Division of Human Living Sciences, Tottori College, Kurayosi, Japan; 2 Department of Life Science and Biotechnology, Faculty of Chemistry, Materials, and Bioengineering, Kansai University, Suita, Japan

**Keywords:** seafood consumption, fish, polyunsaturated fatty acid, health function, cardiovascular disease

## Abstract

In recent years, in developed countries and around the world, lifestyle-related diseases have become a serious problem. Numerous epidemiological studies and clinical trials have demonstrated that diet is one of the major factors that influence susceptibility to lifestyle-related diseases, especially the middle-senile state. Studies examining dietary habits have revealed the health benefits of seafood consumption. Seafood contains functional components that are not present in terrestrial organisms. These components include n-3-polyunsaturated fatty acids, such as eicosapentaenoic acid and docosahexsaenoic acid, which aid in the prevention of arteriosclerotic and thrombotic disease. In addition, seafood is a superior source of various nutrients, such as protein, amino acids, fiber, vitamins, and minerals. This review focuses on the components derived from seafood and examines the significant role they play in the maintenance and promotion of health.

## 1. Introduction

Lifestyle-related diseases, such as obesity, diabetes, hypertension, and hyperlipidemia, are widespread and increasing in developed countries. Metabolic syndrome includes a cluster of symptoms that are related to lifestyle diseases and is associated with an increased risk of type 2 diabetes, some types of cancers (Cerchietti et al., 2007), cardiovascular disease (CVD) (Hwu et al., 2008), and nonalcoholic fatty liver (Byrne 2010). Together with the rapid increase in the number of older people with lifestyle diseases, these have become serious national problems, both medically and financially. Increased dietary sugar and fat promotes obesity and diabetes (Linseisen et al., 2009; Cordain et al., 2005). Soft drink and fast-food consumption is influenced by several factors. Some of these factors include, but are not limited to, food availability, preferences, culture, age, and knowledge of nutrition and health. Reshaping the food environment is a promising new approach to lifestyle-related disease problems (Story et al., 2008; Glanz & Yaroch 2004). Seafood is currently accepted as an essential food for humans (FAO 2010). Seafood is highly regarded for its abundance of high-quality proteins, n-3 polyunsaturated fatty acids (PUFAs), and other nutrients, such as minerals, trace elements, and vitamins (FAO 2010). These nutrients are essential for bodily functions and are beneficial to growth, the brain, and the nervous system; they also have anticancer properties (Liao & Chao 2009). Seafood has helped alleviate food crises in many developing countries, providing a valuable supplement to a diverse and nutritious diet. In recent years, seafood consumption has gradually increased throughout the world (FAO 2010).

In Japan, the consumption of livestock food products, such as dairy products, meats, and their processed foods, have increased. This may lead to an increased incidence of CVD as a result of lifestyle-related diseases, such as hyperlipidemia, atherosclerosis, diabetes, and hypertension (Toshima 1994). Epidemiological and experimental reports have demonstrated a relationship between diet and incidence of CVD (Pereira et al., 2004; Osler et al., 2002). Therefore, dietary therapy is considered the first-choice treatment for arteriosclerotic disease and is recognized as being as important as medical treatment. Many researchers have demonstrated that seafood has nutritional characteristics that maintain and promote health (Mozaffarian & Rimm 2006; Hu et al., 2002). In particular, the health benefits of seafood have principally been associated with high intakes of n-3 PUFAs, such as eicosapentaenoic acid (EPA) and docosahexaenoic acid (DHA) (Dyerberg et al., 1978). Fish oil contains abundant EPA and DHA and is sold as a functional food that can promote superior health. Many other bioactive components derived from seafood are also sold and are under development as functional foods (Harris 2004). Functional food is generally consumed as conventional food that forms a part of the daily diet. Functional food provides basic nutritional functions and reduces the risk of lifestyle-related diseases. Seafood and its derived bioactive components can help improve imbalanced dietary habits and prevent lifestyle-related diseases. In this review, we discuss fish consumption around the world and examine the evidence for the beneficial effects of the various components derived from seafood.

## 2. Fish Consumption around the World and Consumer Demand for Seafood

Over the past 40 years, fish and seafood consumption underwent a major change. In 2008, capture fisheries and aquaculture supplied more than 140 million tons of fish around the world (FAO 2010), approximately 115 million tons of which were for human consumption. Although the estimated per capita supply was approximately 10 kg in the 1960s, by 2008 it had increased to an average of 17 kg (FAO 2010). Adults require 60 g of protein each day; approximately 50% of this amount can be supplied by 150 g of fish. In 2007, fish supplied 15.7% of the animal protein consumed and 6.1% of all protein consumed (FAO 2010). Over just a few years in China, the per capita fish supply increased rapidly and was approximately 26 kg in 2008. Asia accounted for two-thirds of human consumption; 36.9 million tons were consumed outside China and 33.6 million tons were consumed in China (FAO 2010). The average fish consumption per capita for North America, Central America and the Caribbean, South America, Oceania, and Europe was 24.1, 9.5, 8.4, 20.8, 24.5, and 20.8 kg, respectively (FAO 2010). Fish and seafood consumption varied by more than 100-fold between different areas of the world as well as between the inland and coastal regions of countries. Over the past 20 years, the food security of seafood has been improved due to technological developments in processing, distribution, transportation, and storage. These improvements realized cost saving and enhanced safety and quality. Moreover, the development of large-scale, long-distance refrigerated transport and faster shipments revitalized international trade and resulted in the consumption of a wider variety of species and fresh fish. In developed countries, consumers demanded high quality, convenience, reliability, and safety. Consumers in these countries also seek out food that has health-promoting qualities.

## 3. Health Effects of Seafood Consumption

Epidemiological evidence gathered from Greenland Inuit and Japanese fishing villages has demonstrated that the intake of marine animal products is effective in the prevention of CVD (Kagawa et al., 1982; Bang et al., 1980). Many other studies from a variety of countries have also reported that seafood consumption helps protect against lifestyle-related diseases. Numerous epidemiological studies have examined the relationship between dietary marine products and CVD (Guallar et al., 2002; Krauss et al., 2000; von Schacky et al., 1999; Singh et al., 1997). In one report, individuals who consumed fatty fish had a 34% reduction in CVD in a three-cohort study (Oomen et al., 2000), and 35 g/day of fish consumption resulted in decreased CVD mortality (Daviglus et al., 1997).

A meta-analysis revealed that individuals who consumed fish once a week had a 15% lower risk of CVD mortality compared with individuals who consumed no fish (He et al., 2004). The intake of lean and fatty fish in a sample British population was associated with a reduction in diabetes risk in the epidemiological data from the European Prospective Investigation of Cancer (EPIC)-Norfolk cohort study (Patel et al., 2009). One ecological study reported that high frequency fish and seafood consumption decreased the risk of type 2 diabetes in populations with an overweight group (Nkondjock & Receveur, 2003). Sufficient seafood consumption in childhood has been demonstrated to help ensure good fetal neuron development and infant and child cognitive and visual development (Ryan et al., 2010; Carlson, 2009); however, whether or not these positive effects continue into adulthood has not been confirmed. The medical benefits of fish consumption have also been examined as they pertain to inflammatory diseases (Gopinath et al., 2011; Rosell et al., 2009), certain cancers (Szymanski et al., 2010; Dewailly et al., 2003; Zhang et al., 1999), dementia (Cederholm & Palmblad, 2010; Robinson et al., 2010), and psychological status (Appleton et al., 2010).

## 4. The Health Benefits of Bioactive Components Derived from Seafood

The health benefits of seafood and fish oil consumption according to an epidemiological survey of Greenland Inuit by Dyerberg et al are very interesting (Dyerberg et al., 1978). Although the Inuit have a very high-fat diet, the prevalence of ischemic disease is very low in the population. This report received worldwide attention, and studies related to the health functionality of marine products were widely conducted as a result. Many marine organisms inhabit complex environments that are exposed to extreme conditions and, as a result of adapting to the changing environment, they produce a wide range of secondary (biologically active) metabolites. Marine organisms have many bioactive components, such as n-3 PUFAs, protein, fiber, taurine, sterol, and pigments; they also contain unique components that are not present in terrestrial organisms. Nutrients and other bioactive components derived from fish and marine organisms may become functional food ingredients that have medical characteristics and provide health benefits.

### 4.1 n-3 PUFA

The various beneficial effects of seafood have primary been attributed to n-3 PUFAs such as EPA and DHA. Marine organisms have been identified as the only foods that contain a naturally high amount of these fatty acids. This arises from the fact that marine phytoplankton has a high ratio of EPA and DHA, and thus these fatty acids are accumulated in the food chain. The total content of EPA and DHA in fish varies depending on the type of fish and their habitat. The proportion of n-3 PUFAs in fish muscle is higher in fatty fish, such as mackerel, herring, and salmon, than in lean fish, such as cod, haddock, and halibut. In addition, shellfish, such as crab, shrimp, and lobster, have low levels of n-3 PUFAs (Shahidi, 2011).

The metabolites of EPA are the most well known and include eicosanoids, such as the 3-series prostaglandins, prostacyclins, and thromboxanes, and the 5-series leukotrienes (Calder, 1998). The eicosanoids derived from EPA are less active than the pro-inflammatory and pro-thrombotic eicosanoids derived from arachidonic acid. The n-3 and n-6 fatty acids compete for conversion into these important metabolites. In fact, tissue n-6/n-3 levels are largely determined by dietary intake levels (Lands et al., 1992).

Daily intake of n-3 PUFAs such as EPA and DHA reduce the rate of incidence and death from CVD. For example, in the GISSI-Prevention study (Marchioli et al., 2002), more than 2,800 Italians who were heart attack survivors consumed 850 mg of purified EPA/DHA in capsule form for 3.5 years. The results revealed that, compared with a similar number of patients who did not consume the EPA/DHA capsules, there was a 20% reduction in the rate of any-cause death and a 45% reduction in the rate of death from CVD. n-3 PUFAs supplementation therapy continues to demonstrate considerable promise in the primary and secondary prevention of CVD (Lavie et al., 2009).

For preventing general heart disease, the American Heart Association (AHA) recommends approximately 1 g of EPA/DHA per day for coronary heart disease patients (Kris-Etherton et al., 2002). For healthy people, the AHA recommends consuming fatty fish at least twice a week (30–40 g per day), or approximately 500 mg of EPA/DHA per day. Hypertriglyceridemia patients are advised to ingest 2 to 4 g of EPA/DHA per day (Lichtenstein et al., 2006). Further, the consumption of 3 to 20 g or more of EPA/DHA has been examined in terms of the dietary effects on serum triglycerides (TG), blood pressure, platelet aggregation activity, endothelial function, blood vessel flexibility, and inflammation (Kris-Etherton et al., 2003). A previous study found that treatment with 1.5 g of EPA/DHA per day appeared to improve carotid artery plaques stability (Thies et al., 2003). n-3 PUFAs intake has also been associated with beneficial effects related to obesity, insulin sensitivity, and the reduction of inflammatory markers (Ramel et al., 2010; Rudkowska 2010; Ramel et al., 2008; Nettleton & Katz 2005). In a murine model of obesity and insulin resistance, dietary n-3 PUFAs were incorporated in the cell membrane phospholipids; they enhanced the membrane fluidity and the expression, affinity, and some insulin receptors (Das 1999) as well as glucose transporter-4 protein levels in adipose tissue (Peyron-Caso et al., 2002), thereby improving insulin sensitivity.

In addition, n-3 PUFAs have beneficial effects on adipose tissue in obese individuals through reduced body fat mass and stimulated lipid oxidation (Couet et al., 1997), improvement in body weight and satiety regulation (Abete et al., 2010), amelioration of the cytokine profile, including leptin and adiponectin (Abete et al., 2010), and a reduction of inflammation (Das 2005), rheumatoid arthritis (Volker et al., 2000), systemic lupus erythematosus (Walton et al., 1991), Crohn’s disease (Belluzzi et al., 1996), ulcerative colitis (Stenson et al., 1992), and immunoglobulin A nephropathy (Donadio et al., 1994). There is also an increasing amount of evidence that suggests that diets containing fish and/or EPA/DHA may protect against the development of Alzheimer’s disease (Morris et al., 2003) and prostate cancer (Terry et al., 2001).

### 4.2 Phospholipids

Although the majority of fat in seafood is TG, approximately 10% consists of phospholipids (PLs). Numerous studies using animal models have suggested that dietary PLs may be of benefit to human health. For example, phosphatidylcholine, which is a major component of dietary PLs, can decrease blood total lipids (Mastellone et al., 2000) and improve brain function (Chung et al., 1995). Phosphatidylethanolamine and phosphatidylserine can also decrease blood cholesterol (Imaizumi et al., 1983) and improve brain function (Mc Daniel et al., 2003).

As for PLs derived from seafood, EPA and DHA, which have excellent potential as functional food ingredients, are abundant. Dosing with PL-containing n-3 PUFAs resulted in a higher PUFAs content in plasma than dosing with TG-containing n-3 PUFAs (Wijendran et al., 2002; Galli et al., 1992). For these reasons, it is clear that PL-containing n-3 PUFAs are more effective than TG-containing n-3 PUFAs when administered. There have been several human studies that have investigated the beneficial effects of supplementation with dietary krill oil, which has PL-containing n-3 PUFAs rather than TG-containing n-3 PUFAs. Results indicate that krill oil supplementation was well tolerated and caused desirable increases in plasma and cell membrane EPA and DHA levels (Wang et al., 2011; Maki et al., 2009). Furthermore, PL-containing n-3 PUFAs are beneficial in that they can help alleviate obesity-related disorders (Shirouchi et al., 2007) and act as antiinflammatory (Ikemoto et al., 2001), antioxidant (Hiratsuka et al., 2008), and antitumor agents (Hosokawa et al., 2001) in animal experiments. Previous studies have suggested that PL-containing n-3 PUFAs derived from squid mantle muscle decreased serum and liver TG and cholesterol levels compared with that induced by soybean PL- or TG-containing n-3 PUFAs (Hosomi et al., 2010a). Although, research in this field is still in the initial stage, it has been receiving increasing attention as a result of the realization that PL-containing n-3 PUFAs may provide vital outcomes and facilitate progress in the design of beneficial clinical therapies for humans.

### 4.3 Protein, Peptide, and Non-Protein Nitrogen Compounds

It is generally accepted that seafood is a high-quality source of protein and that seafood consumption provides health benefits to growing children, adolescents, and the elderly. Normal dietary habits include fish oil as well as whole fish, which provide many additional nutrients. Dietary n-3 PUFAs decrease serum TG, although they do not lower serum cholesterol (Balk et al., 2006). Therefore, there is a possibility that the health function of fish-based foods is not solely related to EPA and DHA. There is a great deal of research that is focused on the efficacy of EPA and DHA in seafood for human health, whereas there is almost none related to the health effects of proteins. As nutrient components of seafood, the beneficial effect of proteins may have been masked by EPA and DHA in seafood intake intervention studies. Fish protein, which is a major micronutrient in fish, plays an important role in human nutrition worldwide (FAO 2010) and has been used as a main ingredient in processed seafood, such as kamaboko (Japanese fish paste) and fish sausage. Seafood proteins possess excellent amino acid scores and digestibility characteristics. These constitute approximately 10 to 25% of seafoods and can be classified as sarcoplasmic, myobibrillar, and stroma types. In general, amino acid compositions and the bioavailability of animal protein are more suitable than plant protein, and the protein quality of most fish proteins may be equal to that of an ideal protein such as lactalbumin, and exceed that of terrestrial meat (Friedman 1996).

Another aspect of the role of fish proteins in human health pertains to their possible effects on lipid metabolism. In this context, our group and other investigators have demonstrated that fish proteins affect serum cholesterol levels in experimental animals (Hosomi et al., 2009; Wergedahl et al., 2009; Shukla et al., 2006; Zhang & Beynen 1993). A previous study suggested that dietary fish protein decreased serum cholesterol through the inhibition of cholesterol and bile acid absorption and the enhancement of cholesterol catabolism in the liver (Hosomi et al., 2009). In addition, dietary fish protein also has beneficial effects, such as antihypertensive (Boukortt et al., 2004), stimulation of fibrinolysis (Murata et al., 2004), and antiobesity properties (Oishi & Dohmoto, 2009).

In human studies, compared with other animal proteins, dietary cod proteins decreased the highly sensitive C-reactive proteins concentration in serum (Ouellet et al., 2008) and improved insulin sensitivity in insulin-resistant individuals (Ouellet et al., 2007). Recently, the large Nurse’s Health Study, which is a prospective study following more than 84,000 women aged 30 to 55 years over a 26-year period, suggested that increasing the intake of fish as a major dietary protein source provided a significant CVD reduction risk (Bernstein et al., 2010). Thus far, the health functions of various types of fish tissue besides muscle were examined. Although the testes and ovaries are edible parts, only information related to their high cholesterol and nucleic acids content is available. Protamine, which is abundant in fish testes, has been widely used as a pharmaceutical product as an antidote to heparin. It maintains its antihyperglycemic effects together with insulin, and is a natural food preservative. Protamine strongly inhibited the hydrolysis of trioleoylglycerol emulsion using phosphatidylcholine (Tsujita et al., 1996), suppressed lipid absorption in the oral tolerance test in humans (Hoshino et al., 2008), and also suppressed the increase of body mass through the inhibition of fat absorption in small intestine (Duarte-Vázquez et al., 2009). Furthermore, dietary protamine resulted in decreased serum and liver cholesterol levels through the suppression of cholesterol and bile acid absorption, and enhanced the cholesterol secretion from the liver into bile in rats (Hosomi et al., 2010b). In recent years, many people have become interested in the health promotion properties of bioactive peptides prepared from seafood protein.

In a group administered the valyl-tyrosine peptide, which was derived from sardine muscle hydrolysate by alkaline protease, systolic and diastolic blood pressure was reduced by 9.3 and 5.2 mm Hg, respectively, in a four-week double-blind placebo controlled trial (Kawasaki et al., 2000). In addition, the inhibition of lipid peroxidation by a marine bioactive peptide, isolated from jumbo squid, was determined using a linoleic acid model system, and its activity was much higher than α-tocopherol and close to that of butylated hydroxytoluene (Mendis et al., 2005). Marine bioactive peptides also have beneficial effects such as immunomodulating (Duarte et al., 2006), hypocholesterolemia (Wergedahl et al., 2004), and antimicrobial effects (Tincu & Taylor 2004) in animal and in vitro studies. The various health functions of protein and peptides derived from seafood have been clarified by researchers using animal and human studies. Several long-term human studies have been undertaken to evaluate the health effects of marine proteins. In the future, the assessment of the health benefits of marine protein in humans needs to be assessed in long-term clinical trials.

Non-protein nitrogen (NPR) compounds are also present, to various extents, depending on the species. The dark muscles of fish generally contain a higher amount of NPR compounds than the light muscles. NPR compounds in muscle tissues are composed of free amino acids, amines, nucleotides, guanidine and their breakdown products, urea, and ammonium salts (Shahidi, 1998). The contribution of NPR compounds to the taste of seafood is important.

### 4.4 Taurine

With the exception of free amino acid, taurine (2-aminoethanesulfonic acid) is present in nearly all tissues and is particularly abundant in the heart, blood, retina, and developing brain (Wójcik et al., 2010). Taurine synthetic activity in humans is weaker than that in guinea-pigs and rats, and dietary dependence on taurine is high. Hence, taurine is a nonessential but conditionally essential amino acid in the human body (Huxtable, 1992). Taurine has many important roles in several essential biological processes, such as calcium modulation, bile acid conjugation, antioxidation, membrane stabilization, and immunity (Schuller-Levis & Park, 2004; Huxtable 2000; Huxtable 1992). Humans consume taurine largely through seafood, which contains high amounts of taurine compared to meat (Tsuji & Yano, 1984). In particularly, taurine is particularly abundant in some marine invertebrates: oyster tissue has more than 1/100g the taurine content, whereas the taurine content in terrestrial plants is low or absent (Kataoka & Onishi, 1986). Taurine has beneficial antihypertensive (Schaffer et al., 2010; Harada et al., 2004), antihypercholesterolemic (Matsushima et al., 2003), and antiinflammatory effects on lifestyle-related diseases (Jerlich et al., 2000). Furthermore, human intervention studies have revealed that the administration of taurine and n-3 PUFAs has hypolipidemic and antiatherogenic effects compared with n-3 PUFAs supplementation alone (Elvevoll et al., 2008). In non-diabetic obese human subjects, 3 g/day taurine supplementation for 7 weeks reduced serum TG, the atherogenic index, and body weight compared to a placebo group (Zhang et al., 2004). These findings suggest that the consumption of a sufficient quantity of taurine may be important in reducing the risk of lifestyle-related diseases. However, further clinical trials are required to confirm the health promotion mechanism of taurine.

### 4.5 Fiber

In general, muscle-based seafood contains very little carbohydrate and fiber. However, edible seaweed contains a lot of dietary fiber (25–75% dry weight), and water-soluble fiber constitutes approximately 50 to 85% (Jimenez-Escrig & Sanchez-Muniz, 2000). On the basis of their pigmentation, seaweeds are classified into three main groups. Brown seaweeds are predominantly brown due to fucoxanthin and have primary polysaccharides such as fucans, cellulose, alginates, and laminarins (Goni et al., 2002; Haugan & Liaaenjensen, 1994). Green seaweeds are green due to the presence of chlorophyll and ulvan, which is a major polysaccharide component (Robic et al., 2009). Red seaweeds have phycoerythrin and phycocyanin as their principal pigments; they also contain agars and carrageenans as the primary polysaccharides (McHugh 2003). In animal studies, polysaccharides extracted from various edible seaweeds have been found to reduce total cholesterol, low-density lipoprotein (LDL)-cholesterol, and TG in plasma (Amano et al., 2005; Pengzhan et al., 2003). The hypocholesterolemic effect of polysaccharides may be due to an augmented interfering with micelle formation and lipid absorption in the small intestine or an increased excretion of neutral sterols and biliary acids in feces. In addition, sulfated polysaccharides, such as fucoidan and carrageenans, are recognized to possess a number of biological activities, including anticoagulant (Matsubara et al., 2000), antiviral (Artan et al., 2008), antioxidant (Heo et al., 2005), and antiinflammatory (Kim et al., 2009) effects that may have relevance in functional foods, cosmetics, and pharmaceutical applications (d’Ayala et al., 2008; Guo et al., 1998). While a substantial number of studies has been conducted to date both in vitro and in vivo, few studies have been conducted on human subjects. Further study related to the fiber in seaweed should aim to examine the health benefits in human subjects.

### 4.6 Phytosterols

The structure of phytosterols is also similar to cholesterol, with only minor differences in the relative position of ethyl and methyl groups. Phytosterols are common ingredients in plants, and the principal forms are β-sitosterols, stigmasterol, and campesterol. The forms of phytosterols in marine invertebrates include free sterols, stanols, and sterol ester (Kanazawa 2001). Phytosterols are often used to develop health food, including low-fat and fat-free yogurt, milk, juices, spreads, cereals, and bread (Demonty et al., 2009). Clinical trials have consistently shown that an intake of 2 to 3 g/day of phytosterols is associated with a significant lowering (between 4.1 and 15%) of blood LDL-cholesterol (Malinowski & Gehret 2010; de Jong et al., 2008; Patch et al., 2005; Thompson & Grundy 2005). The hypocholesterolemic effects associated with an intake of certain edible microalgae have been demonstrated to be caused by phytosterols, and microalgae have been launched as industrial producers of phytosterols (Plaza et al., 2009; Rasmussen et al., 2009). The lipid-lowering mechanism of phytosterols is thought to occur when phytosterols compete with the absorption of cholesterol by binding to micelles in the intestine (Jones et al., 2000). Their presence in the intestine thus adversely affects the stabilization of cholesterol into micelles, thereby decreasing cholesterol absorption. Another aspect of phytosterols is that they enhance the enterocyte ATP-binding cassette (ABC) G5 and ABCG8 proteins, which act to excrete cholesterol into the intestinal lumen and expression (Marangoni & Poli 2010; Patch et al., 2006). Phytosterols have also been reported to be responsible for other biochemical properties, including antiinflammatory (Houweling et al., 2009), antioxidant (Mannarino et al., 2009), and anticancer effects (Bouic 2001). Few studies have examined the relationship between high-dose phytosterols and the reduction in fat-soluble vitamins, antioxidants, and carotenoids (Musa-Veloso et al., 2011; Katan et al., 2003). Further research is needed to gain more insight into the security of phytosterols as food and functional supplements in the human body.

### 4.7 Carotenoids

Carotenoids are fat-soluble and they have brilliant yellow and orange pigments. They act to transform light energy into chemical energy and antioxidants that inactivate the harmful reactive oxygen species of photosynthetic organisms, plankton, and fungi (Lesser, 2006). One of the most important biological functions of carotenoids such as β-carotene in the human body is their ability to form vitamin A (García-González et al., 2005). However, other carotenoids, such as astaxanthin, lycopene, and fucoxanthin, do not form vitamin A. Recently, the astaxanthin and fucoxanthin derived from seafood have been reported on for a wide range of commercial applications based on their biological properties. Astaxanthin is a xanthophyll carotenoid that is contained in salmonid fish, lobsters, and marine crustaceans. Astaxanthin is considered to have health-promoting effects because astaxanthin oral supplementation in healthy human volunteers caused significant reductions in biomarkers of oxidative stress, inflammation, and hyperlipidemia (Cicero et al., 2007; Karppi et al., 2007; Iwamoto et al., 2000). Non-obsese individuals who consumed astaxanthin for 12 weeks had decreased TG and increased high-density lipoprotein (HDL) cholesterol, which is related to an increase in the adiponectin level (Yoshida et al., 2010). A limited number of clinical studies in humans have been conducted to test the safety of the consumption of astaxanthin. Fucoxanthin is an orange-colored carotenoid found in edible brown seaweeds, such as *Undaria pinnatif*, *Hijiki afusiformis, Laminaria japonica*, and *Sargassum fulvelum* (Maeda et al., 2007). Fucoxanthin prevents the growth of fat tissue, reduces abdominal fat, and reduces the risk of stroke, inflammation, and various cancers (Maeda et al., 2008; Ikeda et al., 2003). Although the beneficial functions of fucoxanthin are beginning to be examined, fucoxanthin administration is known to markedly elevate plasma HDL-cholesterol and total cholesterol levels (Woo et al., 2010; Kadekaru et al., 2008). Before fucoxanthin is used as a functional supplement, further study is required to determine its safety.

## 5. Risk associated with Fish Consumption

The health benefits related to the reduction in risk of CVD have triggered the mass consumption of fish (FAO 2010). Fish consumption, however, also carries certain risks associated with exposure to environmental toxicants. For instance, the only exposure to methylmercury is through edible marine products. Free mercury easily metabolizes methylmercury by microorganisms and is accumulated in the fish at the top of the food chain. Methylmercury exposure affects the highly sensitive nervous system. The developing fetal and infant nervous systems are also highly sensitive to methylmercury. Methylmercury induces central nervous system damage that depends on the amount ingested (Clarkson et al., 2003; Yoshizawa et al., 2002).

Fish consumption recommendations for pregnant women and children are accompanied by warnings regarding how much and what kind of fish should be consumed (FDA 2004). Further, the dioxins and polychlorinated biphenyls contained in seafood have caused concerns related to the health effects of seafood consumption (Arisawa et al., 2005; Arisawa et al., 2003). The balancing of the health benefits and risks of fish intake is an important problem (He, 2009). Some researchers have reported that the consumption of seafood provides benefits that outweigh the risks, except for shark, swordfish, and edible animals and plants from areas with high levels of environmental contaminants (Dewailly et al., 2007; Yaktine & Nesheim 2007; Yoshizawa et al., 2002).

## 6. Conclusion

People have come to realize the importance of seafood in our diet. Numerous studies have proved that some of the best sources of excellent fats, protein, vitamins, and minerals that promote health can be found in seafood. It is unfortunate that it took so many years for the health benefits of seafood to be realized. In the future, an increase in lifestyle-related diseases, the majority of which are a result of dietary habits, is expected in both developed and developing countries (Daar et al., 2007). There is evidence that increased consumption of seafood and bioactive components derived from fish, shellfish, and seaweed could have a positive impact on the health of people around the world. Thus, the role of seafood in the maintenance and enhancement of health may grow stronger, given the problem of lifestyle-related disease and the local food environment. To sum, it is of paramount importance to promote the consumption of seafood and a reduction in high-sugar and high-fat food, including fast food and soft drinks (sugar, in particular), saturated fatty acids, and n-6 PUFAs, which is currently excessive.
